# An innovative approach in the management of alveolar clefts with bone graft harvest from maxillary tuberosity and mandibular wisdom molar odontectomy sites: A case report

**DOI:** 10.1002/ccr3.5253

**Published:** 2021-12-26

**Authors:** Symon Guthua, Peter M. Ng’ang’a, Krishan Sarna, Martin Kamau

**Affiliations:** ^1^ Division of Oral and Maxillofacial Surgery Department of Oral and Maxillofacial Surgery, Oral Pathology and Oral Medicine University of Nairobi Nairobi Kenya; ^2^ Department of Pediatric Dentistry and Orthodontics University of Nairobi Nairobi Kenya; ^3^ Department of Human Anatomy University of Nairobi Nairobi Kenya; ^4^ Present address: Private Practice of Orthodontics Nairobi Kenya

**Keywords:** alveolar bone grafting, alveolar clefts, maxillary tuberosity, odontectomy sites, wisdom molars

## Abstract

Alveolar bone grafting is a complex procedure utilized in alveolar cleft repair; however, the ideal site of bone graft material remains highly debated. In this study, we describe the management of a 14‐year‐old girl with bilateral alveolar clefts using alternative intraoral donor sites for bone graft harvest.

## Graphical abstract

The maxillary tuberosity and odontectomy sites of wisdom molars are overlooked donor sites that may be used in the alveolar cleft reconstruction. A preoperative assessment is recommended to evaluate their viability as potential bone harvest sites.

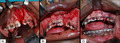

## 1 INTRODUCTION

Alveolar clefts are one of the principal sequelae of cleft lip and palate and are defined as a discontinuity of the dental arch, having an incidence of 0.18–2.50 per 1,000 births.^1^, ^2^ The region of the alveolus most commonly affected lies between the lateral incisor and canine teeth and results in the appearance of a “floating” premaxillary segment if bilaterally present. Alveolar clefts occur in response to divergence from normal embryological development during frontonasal and maxillary prominence growth, contact, and fusion.^3^ A clinical picture comprising of facial growth disturbances, nasal reflux, chronic periodontal inflammation, speech disturbances, and an unsightly esthetic appearance is frequently observed.^4^, ^5^ With recent advancements and innovative techniques in reconstructive surgery aimed at correcting such defects, the quality of life of such patients can be significantly improved.

Alveolar bone grafting (ABG) was described at the beginning of the 20th century as a surgical intervention to be employed in the reconstruction of alveolar clefts.^6^ However, success rates were initially low and tremendously varied depending on the age at which it was performed, the type and source of bone used, and the surgeon's expertise. With the familiarity of the procedure increasing in recent times, ABG is now recognized as the treatment of choice in patients with alveolar clefts secondary to cleft lip and palate.^5^ It remains a complex procedure requiring meticulous planning and flawless execution by a multidisciplinary team partly consisting of craniofacial orthodontists and oral and maxillofacial surgeons.^7^ ABG involves reconstruction of the alveolar defect through the provision of bone (ideally autologous) from a donor site that is then transferred to “fill” in the deficiency at the recipient site. Despite great developments and improvements in the surgical technique, the matter of the ideal source of bone graft material remains controversial to this day.^8^

At present, bone from the iliac crest is widely considered the “gold standard” due to its potential to supply large quantities of endochondral cancellous bone for the reconstruction of large alveolar defects.^9^ However, many authors report significant donor site morbidity, thus leading to the search for alternative intraoral donor sites that have easy accessibility, rapid harvesting time, and low donor site morbidity.^10^ In line with this, intramembranous bone grafts from the mental symphysis and mandibular ramus have been used, albeit sparingly.^5^ Even though endochondral bone grafts remain more popular than intramembranous grafts, the literature reveals that endochondral grafts take a far longer time to achieve complete osseointegration and may postoperatively undergo up to 65% volume loss. In comparison, intramembranous bone grafts have shown promising results with rapid healing, revascularization, and negligible bone loss.^11^

The maxillary tuberosity is recognized as an alternative intraoral donor site; however, its use has been limited to minor maxillary and mandibular alveolar ridge augmentation before fabrication of prosthesis and subantral augmentation.^12^ Bone from this region and other intraoral donor sites develop by intramembranous ossification, similar to the mode of ossification of the alveolar ridge. Indeed, studies on animal subjects show rapid osseointegration and healing when “like is replaced with like.”^13^ A paucity of information exists regarding the use of bone from the maxillary tuberosity in the reconstruction of bilateral alveolar clefts secondary to cleft lip and palate. Additionally, the possibility of odontectomy (disimpaction) sites of the 3rd molars being a donor site for ABG has not been previously discussed. In this study, we report the management of a 14‐year‐old girl presenting with bilateral cleft lip and palate by a combination of orthodontic treatment and surgical reconstruction of the alveolar ridge using bone harvested both from the maxillary tuberosity and odontectomy sites of the 3rd molars.

## 2 CASE PRESENTATION

**FIGURE 1:**
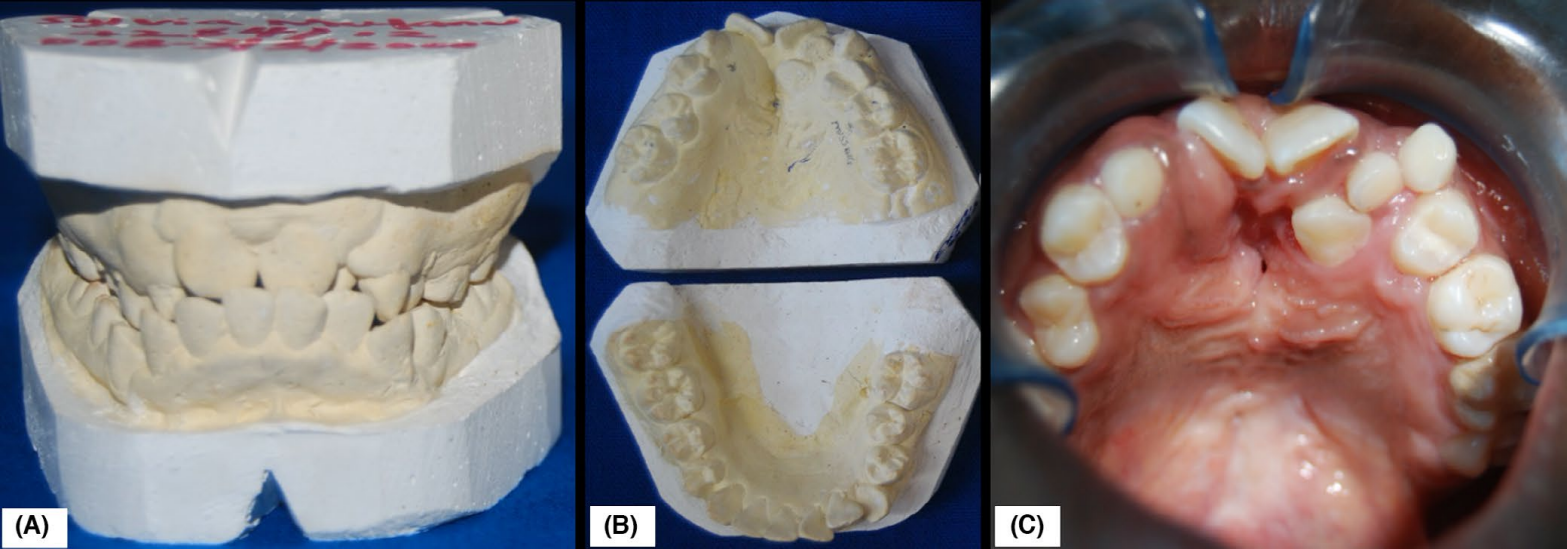
Dental casts showing the maxillomandibular relationship (A) and arch form (B) of the patient and intraoral photograph showing the rotated anterior maxillary segment, anterior crowding, and a centrally placed oronasal fistula (C)

A 14‐year‐old African girl was referred to the Nairobi Hospital, Kenya, seeking treatment for secondary defects of bilateral cleft lip and palate together with oronasal fistulae. The chief complaint was a misalignment of anterior teeth, creating an unsatisfactory esthetic appearance for the patient. In addition, the patient reported difficulty in feeding due to oronasal regurgitation, especially while consuming fluids. The patient had unsuccessfully undergone previous cheiloplasty and several attempts at cleft palate repair before referral. Extraoral examination revealed a whistling deformity characterized by an unsightly central vermillion notching and residual scars on the upper lip consistent with past surgical procedures. Upon intraoral and dental cast analysis, it was verified that the patient had a collapse of the maxilla characterized by a class III skeletal relationship, anterior crossbite, bilateral posterior crossbite, and palatonasal and labionasal fistulae. Additionally, the maxillary lateral incisors (12 and 22), maxillary canine (13), and maxillary 2nd premolar (25) were clinically absent (Figure 1).

**FIGURE 2:**
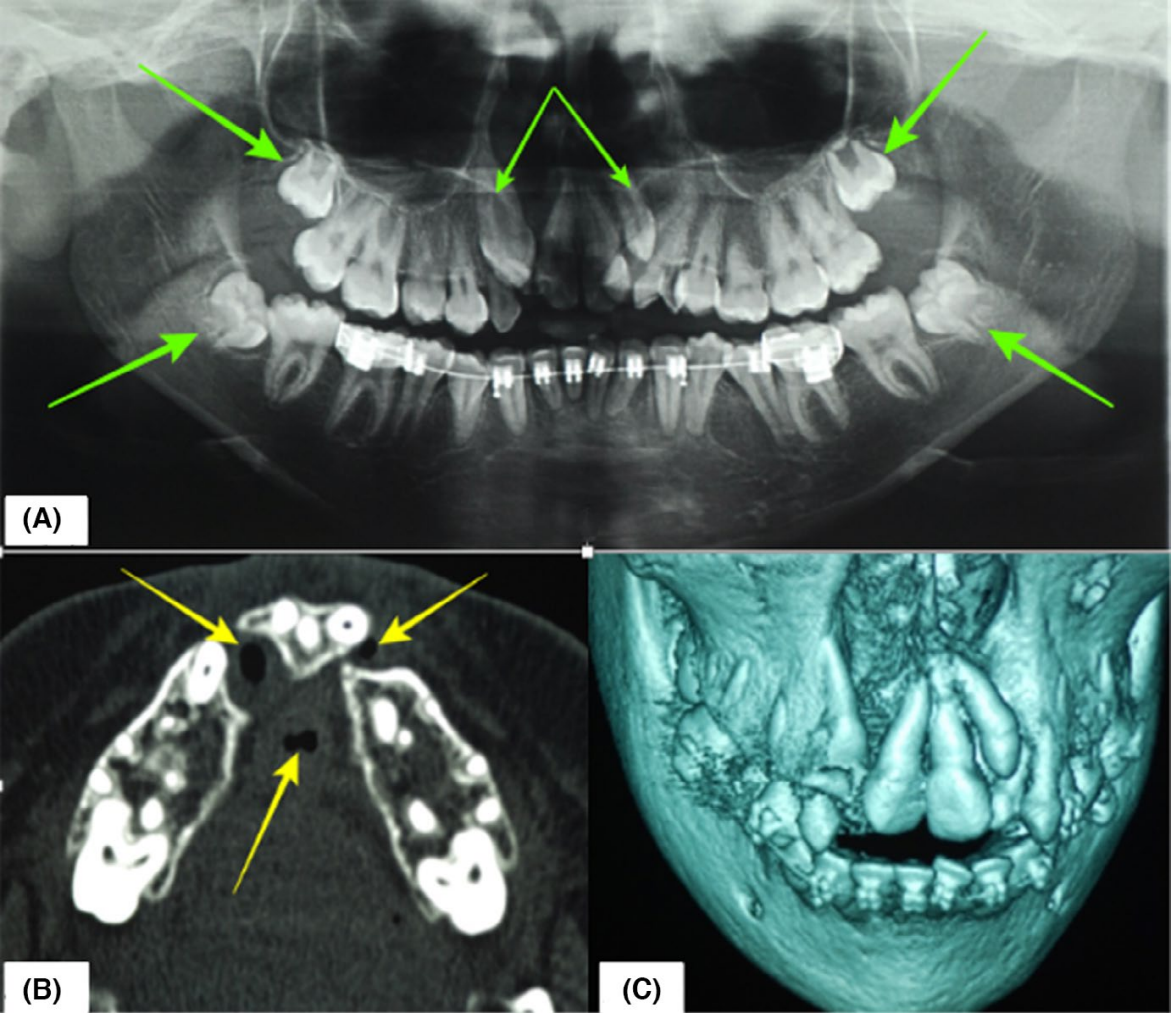
(A) Panoramic radiograph at the beginning of treatment showing anterior maxillary crowding with impacted teeth (green arrows). (B) Axial section CT scan revealing a discontinuity of the maxillary alveolus accompanied by fistulae (yellow arrows). (C) A 3D reconstruction showing the floating anterior maxillary segment

Through the panoramic radiograph, the absence of 12 was confirmed while 13, 18, 22, 28, 38, and 48 were all impacted. A computed tomography (CT) scan revealed a bilateral discontinuity of the maxillary alveolar ridge resulting in a floating anterior maxillary segment with attachment solely to the nasal septum. In addition, a total of three round/oval oronasal fistulae were visualized (Figure 2).

**FIGURE 3:**
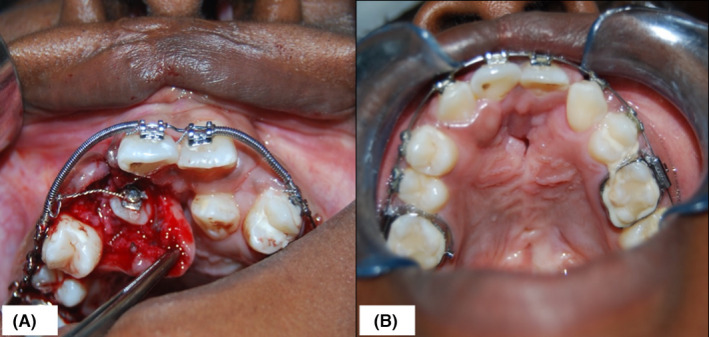
Surgical exposure of 13 and orthodontic traction in order to align the tooth in occlusion (A). Results by the end of phase one treatment (B)

The patient was managed with a multidisciplinary approach in three well‐defined phases. The first phase consisted of presurgical orthodontic treatment and involved the use of a hyrax rapid maxillary expander. The screw was turned one‐quarter of a turn once a day for a total of 5 weeks. At the end of the expansion, the device was kept in place for another 5 months, after which, upper and lower orthodontic fixed appliances were bonded. The lower 1st premolars were extracted in an attempt to balance the occlusion. The final step in the first phase of treatment involved right maxillary ABG to reconstruct the cleft of the alveolus. Surgical exposure and consequent orthodontic traction were then employed to align the 13 into occlusion (Figure 3).

**FIGURE 4:**
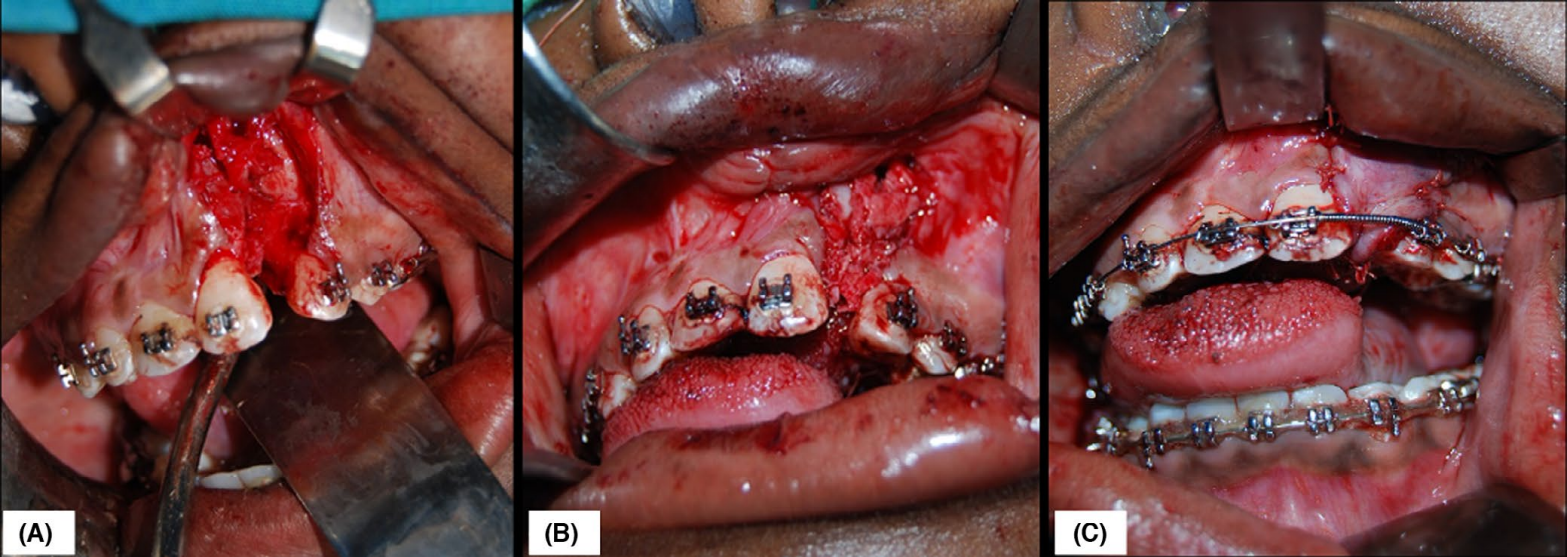
Intraoperative photographs showing the exposure of the cleft segment and soft tissue repair of the nasal floor (A) followed by packing of the bone graft to reconstruct the alveolus (B). This was followed by adequate soft tissue cover using a mucoperiosteal flap (C)

**FIGURE 5:**
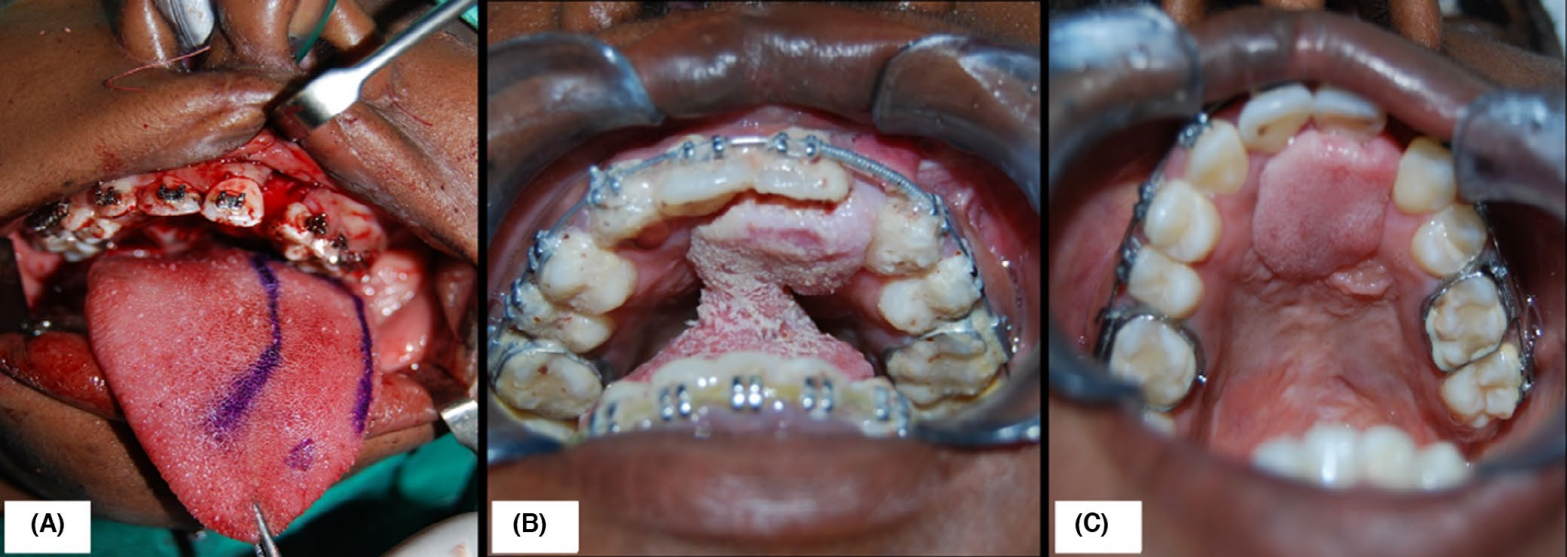
The anteriorly based, left dorsal tongue flap design (A). Suturing of the flap to the palate with adequate pedicle length while its base remained attached to the tongue, promoting vascularization and healing (B). Adequate closure of all oronasal fistulae was achieved with no further complications (C)

**FIGURE 6:**
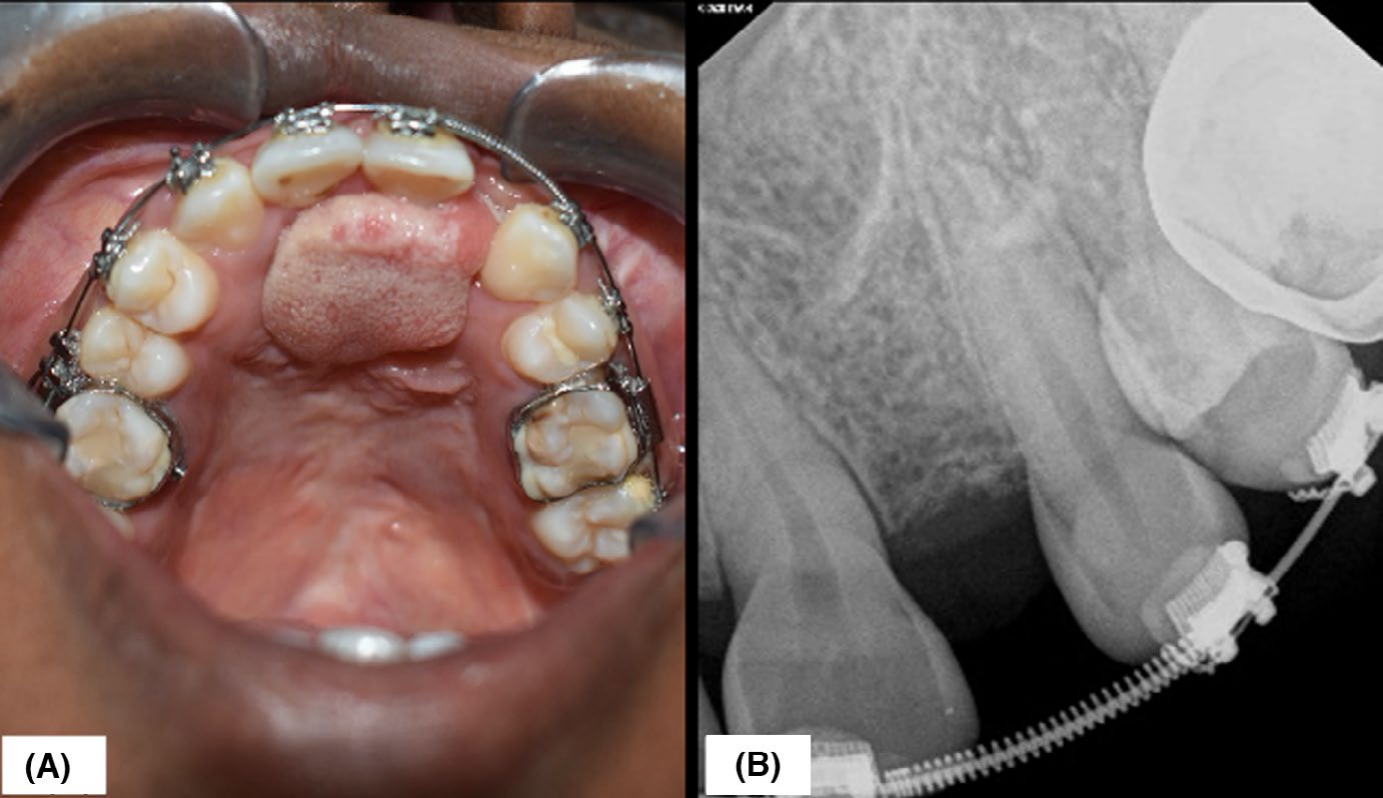
Patient's final intraoral appearance, after completion of treatment (A). The IOPA shows complete osseointegration at the recipient site with adequate bone stock between 21 and 23 (B)

The second phase of treatment entailed left maxillary ABG and closure of the oronasal fistulae. It was noted that the wisdom teeth were impacted (see Figure 2), and upon recommendation from the orthodontist, they were removed. Hence, proper planning of the surgery was imperative, which comprised of surgical odontectomies (disimpactions) of 18, 28, 38, and 48 followed by harvesting of the particulate corticocancellous bone from the maxillary tuberosity, distal to 18 and 28 and from the retromolar area distal to 38 and 48. The volume of bone harvested in this case was 15 cc in total. After the bone was obtained, a buccal flap was raised to expose the cleft region, followed by disimpaction of the unfavorably positioned 22 presents within the cleft. Nasal floor soft tissue repair was then performed followed by packing of the particulate bone into the cleft (Figure 4). Due to the heavy scarring resulting from multiple unsuccessful palatal surgeries previously performed, a poor soft tissue profile (deficiency) was noted around the oronasal fistulae (Figure 3b). This prevented adequate local soft‐tissue closure necessitating the use of an anteriorly based, left dorsal tongue flap. The flap was designed and elevated with a 5 mm thickness and adequate pedicle length that was enough to allow suturing to the palate without any tension. Postoperatively, the patient was fed via nasogastric tube for 5 days after which the oral feeding resumed albeit, on a pureed (blenderized) diet. Three weeks later, the flap was divided and the rest returned to the donor site (Figure 5). Postoperative pain was managed using a combination of paracetamol and diclofenac. The antibiotic cover consisted of augmentin 1.2 g, IV, for 3 days and then 1g peroral twice a day for 4 days. Clinical evaluation after discharge was undertaken at durations of 2 weeks, 1, 3, and 6 months. To assess graft survival and dental arch stability, intraoral periapical (IOPA) and a digital orthopantomogram (OPG) were taken after 6 months (Figure 6).

The third phase of treatment comprised of postsurgical orthodontics to close spaces and coordinate the occlusion. Overall, the orthodontic treatment took 47 months to complete.

## 3 DISCUSSION

Cleft lip and palate are considered the most prevalent congenital craniofacial birth defect and are the second most common congenital malformation of the human body, second only to clubfoot.^14^ Fusion of several structures and processes of the neonatal face result in the development of both the lip and palate between the 4th and 12th week of gestation. A failure of fusion due to genetic or environmental causes may lead to the development of cleft lip and palate.^15^

Alveolar bone grafting forms a fundamental component of the treatment protocol of alveolar clefts in patients with cleft lip and palate. The main objectives of ABG are to establish continuity of the dental arch, facilitate closure of oronasal fistulae, correct the nasal alar bases, and provide solid bone for tooth migration and dental implant placement. Although its use has increased, certain aspects of the surgical technique are shrouded in controversy.^5^ The timing at which ABG is performed is one such dilemma with two possible approaches having been proposed: primary bone grafting during infancy or secondary bone grafting during the mixed dentition period.^16^ Recently, some consensus seems to have been reached with most surgeons opting for secondary ABG between 8 and 10 years of age due to lower incidences of complications such as maxillary growth restriction, which have been frequently reported after primary ABG.^8^, ^17^ However, the current debate revolves around the choice of an ideal source of bone graft material, which may be even more controversial than the timing issue of ABG.

The ideal bone graft sites can be grouped into either extraoral sites such as the iliac crest, proximal tibia, and ribs or intraoral sites such as the mandibular symphysis and mandibular ramus.^5^ The selection of a particular donor site is dependent upon the size of the defect being repaired, ease of harvest, donor site morbidity, and the experience and preference of the surgeon.^10^ The various donor sites also provide the surgeon with a choice of either endochondral cancellous bone (extraoral sites) or intramembranous corticocancellous bone (intraoral sites).^8^ For many years, it has been believed that endochondral bone is far more superior to the intramembranous bone.^8^, ^10^ However, due to the increased cortical bone content in intramembranous bone harvested from intraoral sites, it undergoes delayed resorption and, therefore, maintains its volume for a prolonged period compared to endochondral bone.^13^ Additionally, intramembranous grafts have been shown to develop up to 166% more new bone around the graft site, which is significantly higher than endochondral grafts.^11^

The maxillary tuberosity contains an appreciable amount of intramembranous bone, which can be used to reconstruct small to medium alveolar clefts. After a careful patient assessment, the amount of bone obtained can be enhanced further by also harvesting bone from odontectomy sites of the wisdom molars. If all these sites are utilized, there is a potential of harvesting up to 30 ccs of bone, which can satisfy extensive grafting requirements.^18^ Other salient advantages of using these sites as a source of bone graft lie in their convenient anatomical location, a single surgical site in the same region of the body as opposed to two sites away from one another, minimal postoperative complications, hidden scars, and a much shorter hospital stay.^5^

We, therefore, strongly recommend that clinical examination of these regions be a part of the routine evaluation of patients when selecting a donor site for ABG.^12^ During the preoperative assessment, cone beam computed tomography (CBCT) can be implemented to make an accurate three‐dimensional analysis of the maxilla and mandible for the best sites of bone graft material. Additionally, the timing of removal of the wisdom teeth should ideally coincide with the repair of the clefts. It seems that the use of these sites can be a simple and valuable alternative technique for alveolar cleft reconstruction with fewer intraoperative difficulties and postoperative complications. If the maxillary antrum is exposed during bone harvesting, a primary immediate repair can be performed.^18^ Since some of these patients present after having undergone unsuccessful repair of oronasal fistulae, advancement of local flaps to close the defects will not be successful. A well‐designed pedicle tongue flap is the best alternative for soft tissue repair of such defects.

In the present case, ABG was successful based on the clinical and radiographic findings (Figure 6). There was the establishment of a good maxillary arch form with stabilization of the premaxillary segment. There was also complete closure of oronasal fistulae, and significant improvement in the patient's occlusion and facial profile. Overall, a satisfactory esthetic outcome was achieved.

## 4 CONCLUSIONS

Alveolar bone grafting is a complex procedure requiring interdisciplinary effort to achieve the intended results. We have presented a case where ABG was performed using unconventional donor sites such as the maxillary tuberosity and mandibular wisdom molar odontectomy sites to harvest significant amounts of bone. The intramembranous bone harvested from these sites achieved complete osseointegration with minimal resorption after grafting. Therefore, these could be identified as alternative donor sites during the preoperative assessment of patients requiring ABG secondary to cleft lip and palate. Furthermore, where there is immense scarring making local tissues unsuitable for oronasal fistula repairs, a tongue flap may be a good alternative to close the fistulae.

## CONFLICT OF INTEREST

The authors declare that there is no conflict of interest.

## AUTHOR CONTRIBUTIONS

Symon Guthua, Peter M. Ng'ang'a, and Martin Kamau performed all clinical procedures described in this manuscript. Krishan Sarna acquired patient data and drafted the manuscript while being supervised by all authors who approved the final version.

## CONSENT

Written informed consent was obtained from the patient's parents to publish this report following the journal's patient consent policy.

## Data Availability

The data that support the findings of this study are available on request from the corresponding author. The data are not publicly available due to privacy or ethical restrictions.
